# CRABP2 promotes metastasis and lipid droplet accumulation in non-small cell lung cancer by downregulating PLAAT4

**DOI:** 10.7150/jca.112019

**Published:** 2025-06-23

**Authors:** Jie Xia, Bi Peng, Jianhua Wang, Fang Li, Guoxian Long

**Affiliations:** 1 Department of Respiratory and Critical Care Medicine, Tongji Hospital, Tongji Medical College, Huazhong University of Science and Technology, Wuhan, China.; 2 Department of Oncology, Tongji Hospital, Tongji Medical College, Huazhong University of Science and Technology, Wuhan, China.

**Keywords:** Non-small cell lung cancer, accumulation of lipid droplets, metastasis, CRABP2

## Abstract

Non-small cell lung cancer (NSCLC) is a highly prevalent and aggressive cancer with a high incidence. While cellular retinoic acid binding protein 2 (CRABP2) has been implicated in tumor progression, metastasis and drug resistanceacross multiple cancer types, its functional role and molecular mechanisms of CRABP2 in NSCLC progression remain largely unexplored. In this study, we demonstrated that CRABP2 expression was significantly elevated in NSCLC tissues compared to adjacent normal tissues, and high levels of CRABP2 correlated with reduced overall survival. Functionally, knockdown of CRABP2 inhibited NSCLC cell proliferation, migration, and invasion, and lipid droplet accumulation *in vitro*, while CRABP2 targeting inhibited tumor growth, lipid droplet content and metastasis in xenograft model. Mechanistically, CRABP2 was identified to bind to Phospholipase A/acyltransferase 4 (PLAAT4) and decreases its protein stability. Notably, inhibition of PLAAT4 reverses the shCRABP2-induced suppression of malignant phenotypes and lipid droplet formation. our findings reveal a novel CRABP2/PLAAT4-mediated lipid metabolic axis drives NSCLC progression and metastasis. These findings suggest that targeting CRAPB may offer a novel approach to therapeutic intervention for NSCLC.

## Introduction

Lung cancer remains the foremost cause of cancer-related mortality worldwide, particularly among men, where it ranks as the leading type, and second among women 1, 2. Non-small cell lung cancer (NSCLC) represents approximately 80% to 85% of all lung cancer cases, with adenocarcinoma being the most prevalent subtype. Most patients usually have a better prognosis when they receive treatment in the early stages; however, once the cancer has spread (usually in stages III or IV), the disease progresses rapidly 3. Unfortunately, the limited understanding of the mechanisms underlying cancer progression and metastasis has resulted in few effective treatment options for advanced stages. This situation has created barriers to improving treatment outcomes and underscores the importance of deeply researching the mechanisms of lung cancer progression.

Metabolic reprogramming represents a fundamental hallmark of cancer, equipping tumor cells with the energy and diverse metabolites necessary for sustaining their aberrant survival and proliferation 4. Beyond the well-documented alterations in glucose metabolism, enhanced lipid metabolism emerges as a significant characteristic of cancerous metabolic profiles 5. Rapidly proliferating cancer cells undergo a notable shift in lipid metabolism, leading to a marked increase in lipid accumulation. This upregulation can occur through heightened activation of endogenous synthesis or increased uptake of exogenous lipids and lipoproteins 6. Lipid metabolism plays a significant role in the growth and progression of various cancer types, including lung cancer 7-10. The expression levels of lipid metabolism-related enzymes, such as fatty acid synthase (FASN), acetyl-CoA carboxylase (ACC), and acetyl-CoA synthetase (ACSS2), are frequently elevated in lung cancer. Increased expression of these enzymes facilitates fatty acid synthesis and metabolism, thereby promoting tumor growth and survival 11-14. Therefore, therapeutic strategies that target lipid metabolism may offer potential benefits in the management of lung cancer.

Cellular retinoic acid binding protein 2 (CRABP2) belongs to the retinoic acid binding protein family and the lipid calcium-binding protein/cytosolic fatty acid binding protein family, and mainly participates in the signal transduction of retinoic acid 15. Its expression levels have been found to be abnormal in various cancers, and plays a pivotal role in tumor progression and drug resistance. 16-19. For example, Feng X et al. 20 demonstrated that CRABP2 inhibits invasion and metastasis in estrogen receptor-positive (ER+) breast cancer, while promoting these processes in estrogen receptor-negative (ER-) breast cancer. In gastric cancer, CRABP2 has been shown to contribute to oxaliplatin resistance by enhancing BAX degradation, thereby reducing apoptosis 21. Furthermore, in thyroid cancer, CRABP2 expression correlates with unfavorable prognostic outcomes and is linked to enhanced migratory and invasive capabilities 22. Notably, CRABP2 levels are significantly elevated in lung adenocarcinoma tissues and plasma, correlating with poor outcomes for patients 23, 24, suggesting a potential role in the progression of this malignancy. However, the functional role and molecular mechanisms of CRABP2 in NSCLC remain to be fully elucidated.

This research revealed a connection between CRABP2 and the progression and lipid metabolism of NSCLC. At the same time, inhibiting CRABP2 expression can significantly reverse lipid metabolism and metastasis. Mechanistically, we found that CRABP2 can bind to PLAAT4, and the knockdown of CRABP2 causes upregulation of PLAAT4 expression, leading to an overall decrease in lipid metabolic activity, ultimately inhibiting NSCLC cell growth and metastasis. This study offers a novel perspective on understanding NSCLC cell growth and metastasis and provides experimental evidence for potential clinical implications.

## Materials and methods

### Data Sources

The UALCAN database (https://ualcan.path.uab.edu/index.html) was utilized to screen for the upregulated gene set in the lung adenocarcinoma (LUAD) dataset and analyze the expression levels of the CRABP2 gene in LUAD. The Clinical Proteomic Tumor Analysis Consortium (CPTAC, https://ualcan.path.uab.edu/analysis-prot.html) dataset was employed to examine CRABP2 protein expression in LUAD, as well as its levels across various tumor grades. Using Gene Expression Profiling Interactive Analysis (GEPIA) web server, overall survival (OS) analysis was conducted based on the expression of CRABP2 in LUAD, and the Mantel-Cox test was used for hypothesis testing. The cohort threshold was set at 50%.

### Clinical specimens and Data Collection

All fresh NSCLC specimens (20 lung adenocarcinoma tumors and paired adjacent normal tissues) were prospectively collected from treatment-naïve patients undergoing curative resection at the Oncology Department of Tongji Hospital. Following excision, specimens were placed in cryogenic tubes and promptly stored in liquid nitrogen for preservation. The inclusion criteria include: (1) patients aged 18 years or older, (2) a confirmed diagnosis of lung adenocarcinoma through pathological examination. The exclusion criteria included: 1) prior neoadjuvant therapy (chemotherapy/radiotherapy), 2) concurrent malignancies or metabolic disorders (e.g., diabetes, dyslipidemia), 3) incomplete clinicopathological documentation. All clinical samples were obtained in compliance with the regulations set forth This study was approved by the Ethics Committee of Tongji Hospital (No. TJ-IRP20230518), and conducted in accordance with the Declaration of Helsinki. Written informed consent was obtained from each participant for this study.

### Cell culture

Human normal lung epithelial cell line BEAS-2B was obtained from NCACC cell bank (Shanghai, China) and NSCLC cell lines (A549, H1650, H1299, H358, and H1563) were all obtained from Pricella (Shanghai, China). BEAS-2B cells were cultured in BEGM BulletKit medium, the A549 cell line was cultured in Ham's F-12K medium containing 10%FBS, and H1650, H1299, H358, and H1563 cell lines were cultured in 1640 medium containing 10% FBS, respectively. The cell culture environment was maintained at 5% CO_2_ and 37°C.

### Colony formation assay

Cells are digested with trypsin and suspended in culture medium, counted, and plated onto 6-well plates (1000 cells per well). The cells are cultured at 37°C and 5% CO_2_ for 14 days. The cells are washed with PBS twice. They are then fixed with 4% paraformaldehyde and stained with 1% crystal violet for 30 minutes. The clones are photographed and counted.

### Wound healing assay

Cells were digested with trypsin and resuspended in serum-free culture medium, counted, and adjusted to a density of 1×10^6 cells/mL before being plated into 6-well plates (2 mL of cell suspension per well). The cells were incubated at 5% CO_2_ and 37°C until they reached approximately 95% confluence. Subsequently, wounds were made using 10 μL pipette tips. The cells were washed three times with PBS to remove any suspended cells and then cultured in serum-free RPMI-1640 medium. Images of cell migration were captured at time points of 0 h, 12 h, 24 h, and 48 h post-scratching, ensuring consistent fields of view for all images. Scratch images were analyzed using Image J software.

### Transwell assay

Matrigel matrix was diluted to a 1:6 ratio in serum-free RPMI-1640 medium. Each well of the culture plate was added with 30 μL of the above mix and incubated in a 37°C incubator for 4-5 hours. After digestion, cells were suspended in serum-free RPMI-1640 medium and diluted to 4×10^4 cells /100μL. Then, 100 μL of cell suspension was added to the upper well, and 600μL of RPMI-1640 medium containing 10% FBS to the lower well. The chamber was cultured in a CO_2_ incubator for 24 h, and then the medium in the transwell chamber was discarded. Cells were fixed in methanol for 30 min and then were stained in 0.1% crystal violet for 30 min at room temperature. After washing twice in PBS, the remaining cells on the upper layer of the chamber were gently wiped off with a cotton swab. At least four fields were randomly selected under the microscope to count the number of migrating and invading cells, with 3 replicates in each group.

### BODIPY staining

BODIPY staining was conducted to assess the content of lipid droplets. In brief, the NSCLC cells were fixed with 4% paraformaldehyde and incubated with BODIPY-C16 (20 μg/mL) at 37°C for 20 min. Following this, the cells were counterstained with DAPI for 2 min. After being washed with PBS three times, the fluorescent signals were observed under a fluorescence microscope (Olympus).

### Construct the protein-protein interaction (PPI) network

To construct the PPI network, we first retrieved the intersection targets from the String database (https://string-db.org), specifically for “Homo sapiens,” and set a moderate confidence threshold of 0.400. Additionally, we obtained the PPI structure for CRABP2 and imported it into Cytoscape for graph optimization. R software was then utilized to create a bar chart illustrating the top 30 core genes.

### Co-immunoprecipitation (Co-IP)

To assess the endogenous interaction between CRABP2 and PLAAT4 proteins, cells were lysed using an IP lysis buffer containing a mixture of protease inhibitor. The cell lysate was pre-washed with protein A/G beads at 4°C for 4 h, followed by immunoprecipitation utilizing anti-CRABP2 or anti-PLAAT4 antibodies coupled to protein A/G at 4°C overnight. Finally, the protein A/G beads capturing the target proteins were cleaned with IP lysis buffer and analyzed by Western blot.

### Animal experiments

For *in vivo* xenograft assay, A549 cells were cultured and treated the indicated constructs: Lv-NC, Lv-sh-CRABP2, and Lv-sh-CRABP2+sh-PLAAT4. Subsequently, these treated cells (1 × 10^6^ cells) were subcutaneously injected into the dorsal flanks of 5-week-old male BALB/c nude mice (Gempharmatech, Nanjing, China), with 6 mice per group. Tumor size was measured every week for 4 weeks. The tumor volume (V) was calculated by the formula (length × width × width)/2. The tumors were excised and embedded in paraffin. For lung metastasis assay, the indicated A549 cells were treated as indicated and injected into nude mice via tail vein injection (n = 6 mice). Two months post-injection, the mice were euthanized, and their lungs underwent formaldehyde fixation followed by Histopathological examination. This study was approved by the Ethics Committee of Tongji Hospital (No. TJ-IRP20230518).

### Cholesterol levels in tissue

Tissue samples were mechanically homogenized in an ice-water bath. The homogenate was then centrifuged at 2500 rpm for 10 min, and the supernatant was collected for further analysis. According to the instructions provided with the Total Cholesterol Assay Kit (Nanjing Jiancheng Bioengineering Institute, China), the collected supernatant was mixed with reagents and incubated at 37°C for 10 min. The absorbance of each well was measured at 500 nm using a microplate reader. Meanwhile, the protein concentration of the homogenate was determined using the Total Protein Quantitative Assay Kit (Nanjing Jiancheng Bioengineering Institute, China).

Cholesterol content (mmol/gprot) =

[(A_sample_-A_blank_)/(A_standard_-A_blank_)]*C_standard_÷Cpr

### Oil Red O staining

Frozen sections of xenograft tumors were fixed in a tissue fixative for 15 minutes, rinsed with tap water, and air-dried. The sections were stained with Oil Red O staining solution for 8-10 min. Then, the sections were immersed in 60% isopropanol for differentiation. Subsequently, the sections were immersed in distilled water for 10 seconds. Following this, the slices were counterstained with hematoxylin for 3-5 min, washed in distilled water, and immersed in differentiation solution for 5 s. Finally, the slides were sealed using a glycerol-gelatin mounting medium. Microscopic examination included image acquisition and analysis. The lipid droplets were orange to bright red, and the nuclei were blue.

### Statistical analysis

GraphPad Prism version 8.0 was used for data analysis and graphical presentation. All experiments were conducted at least three times. Quantitative data are expressed as mean ± standard deviation (SD). Statistical comparisons between two groups were performed using the Student's t-test, while comparisons involving three or more groups were analyzed using one-way or two-way ANOVA followed by Tukey's post-hoc test. A p-value of less than 0.05 was considered statistically significant.

## Results

### CRABP2 is highly expressed in NSCLC and correlated with poor prognosis

**Figure 1:**
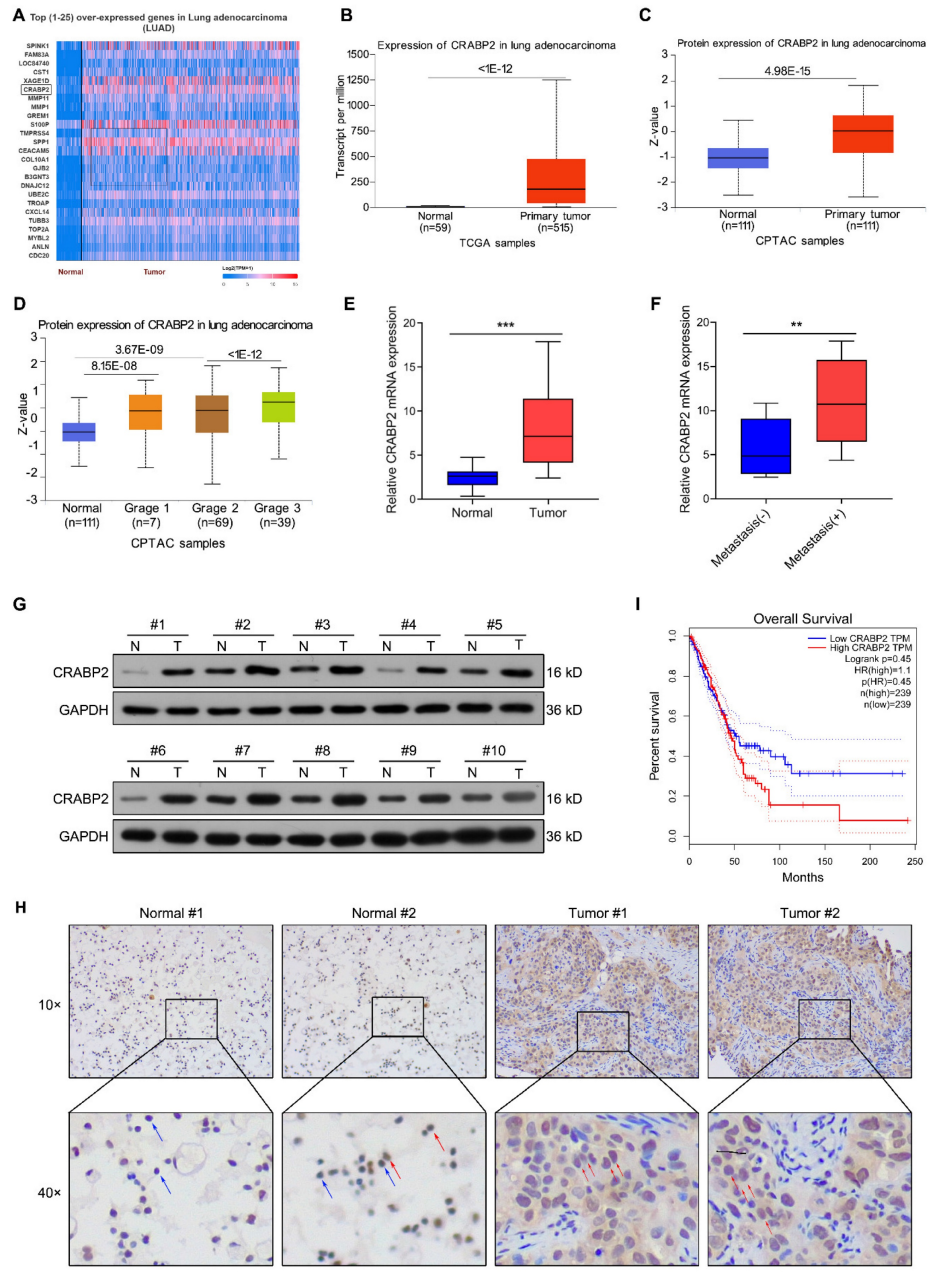
The elevated expression of CRABP2 in NSCLC and its association with unfavorable prognosis. (A) Top (1-25) over-expressed genes in LUAD identified through bioinformatic analysis via the UALCAN database. (B) Comparison of CRABP2 transcript levels in LUAD versus normal samples via the UALCAN database. (C) Comparison of CRABP2 protein expression in LUAD versus normal samples via the CPTAC database. (D) Analysis of CRABP2 protein expression across different tumor grades of LUAD via the CPTAC database. (E) Quantitative RT-PCR assessed CRABP2 mRNA expression in 20 pairs of NSCLC cancerous and adjacent non-cancerous specimens. *** *P* < 0.001. (F) CRABP2 mRNA expression in NSCLC tissues with or witout metastasis. ** *P* < 0.01. (G) Western blotting analysis of CRABP2 protein expression in 10 pairs of NSCLC cancerous and adjacent non-cancerous specimens. (H) Immunohistochemical staining of CRABP2 in NSCLC cancerous and adjacent non-cancerous specimens. The red arrows indicate positive cells, and the blue arrows indicate negative cells. (I) Kaplan-Meier survival analysis was conducted to evaluate the impact of CRABP2 expression on the overall survival of LUAD patients was evaluated using data sourced from the GEPIA database.

We conducted bioinformatic analysis of lung adenocarcinoma (LUAD) tissues via the UALCAN database and Top (1-25) over-expressed genes in LUAD were shown in Fig. 1A. The analysis revealed a significant increase in the expression of CRABP2 transcript in LUAD tissues compared to normal tissues (Fig. 1B). Data from CPTAC indicated that the protein expression of CRABP2 were also elevated in cancerous tissue from 111 LUAD patients when contrasted with adjacent non-tumor samples (Fig. 1C). Furthermore, the expression of CRABP2 protein in Grade 3 was higher than in Grade 2 (Fig. 1D). After that, the expression level of CRABP2 was determined in NSCLC tissues. RT-PCR results demonstrated that overall CRABP2 levels were significantly higher in cancerous tissues relative to adjacent normal tissues (Fig. 1E). Notably, in the analysis of metastasis characteristics, the expression level of CRABP2 in metastasis-positive tumor tissues was 1.9 times higher than that in non-metastatic groups (*P* = 0.009, Fig. 1F). The chi-square test results in Table 1 indicated that although the expression level of CRABP2 was not statistically significantly associated with the metastasis status of lung cancer patients (p = 0.074), the upregulated expression trend in the metastasis group suggested that it might be involved in the biological process of tumor progression and metastasis. In addition, randomly selected 10 pairs of NSCLC tissue samples underwent Western blot analysis for protein levels. Figure 1G shows that CRABP2 protein levels were elevated within tumor specimens compared to adjacent normal tissues. Furthermore, Immunohistochemical staining further confirmed high expression of CRABP2 within tumor cells, showing notable cytoplasmic accumulation (Fig. 1H). Lastly, Kaplan-Meier survival analysis indicated that patients exhibiting high CRABP2 levels had reduced overall survival times compared to those with lower expressions based on data from the GEPIA database (Fig. 1I).

### Knockdown of CRABP2 inhibits tumourigenic properties of NSCLC cells

**Figure 2:**
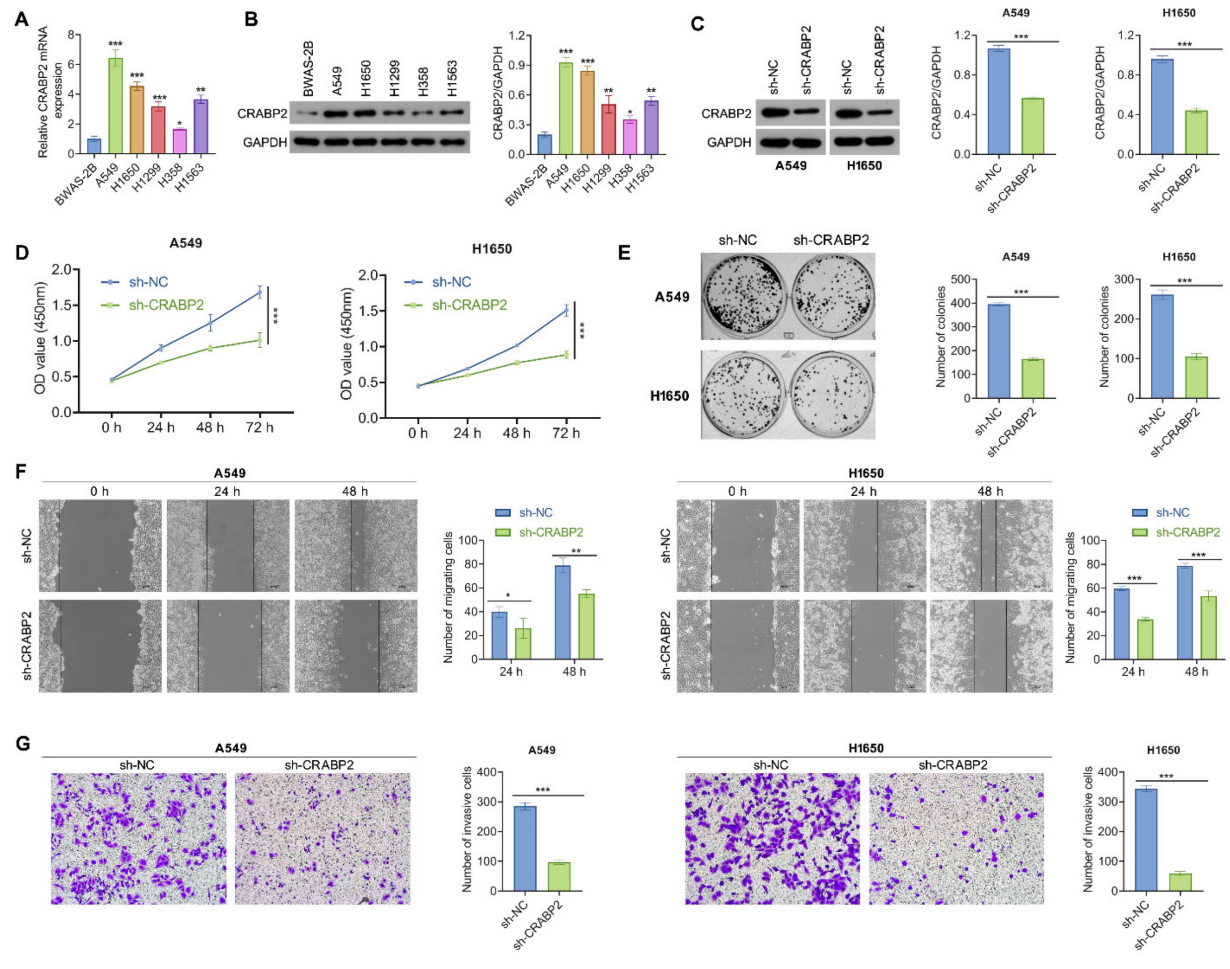
Downregulate of CRABP2 inhibits NSCLC cell proliferation, migration, and invasion. (A) Expression of CRABP2 mRNA in human normal lung epithelial BEAS-2B cells and five NSCLC cell lines was determined by quantitative real-time PCR analysis. (B) Protein expression of CRABP2 in five NSCLC cell lines and BEAS-2B cells was determined by western blot analysis. (C) Western blot analysis of CRABP2 protein expression in stable CRABP2-knockdown (shCRABP2) A549 and H1650 cells via lentiviral transduction. (D) Cell viability of A549 and H1650 cells following CRABP2 knockdown using CCK-8 assay (n = 3). (E) Colony formation assays in A549 and H1650 cells with CRABP2 knocked down (n = 3). (F) The migration ability of A549 and H1650 cells following CRABP2 knockdown using Wound healing assays (n = 3). (G) Matrigel invasion assays were conducted to assess the invasion ability in A549 and H1650 cells with CRABP2 knocked down (n = 3). *P < 0.05, **P < 0.01, ***P < 0.001.

We further explored the function of CRABP2 in tumorigenic characteristics of NSCLC cancer cells. First, we observed that A549 and H1650 cells exhibited the highest expression of CRABP2 among several NSCLC cell lines (Figs. 2A-B).

Subsequently, stable CRABP2 knockdown (shCRABP2) was established in A549 and H1650 cells through lentiviral transduction, with successful validation of CRABP2 expression via Western blot analysis (Fig. 2C). The reduction of CRABP2 led to a marked decrease in cell viability for both A549 and H1650 cells (Fig. 2D). This suppressive effect was further corroborated by colony formation assays, which demonstrated that knockdown of CRABP2 significantly reduced colony formation compared to control groups in both A549 and H1650 cells (Fig. 2E).

Additionally, the wound healing assay results indicated that NSCLC cells infected with shCRABP2 lentivirus showed a reduced ability to close wounds compared to control cells, approximately 1.35 times lower than the controls (Fig. 2F). Following this, Matrigel invasion assays were conducted to evaluate their invasive capabilities. Microscopic images and corresponding histograms (Fig. 2G) revealed that the number of CRABP2-knockdown NSCLC cells migrating through Matrigel to the lower chambers was significantly reduced compared to control cells, indicating a decreased invasive ability upon CRABP2 knockdown in NSCLC cells. These findings collectively indicate that CRABP2 knockdown attenuates both proliferation and metastatic potential *in vitro* for NSCLC cells.

### Downregulate of CRABP2 inhibits lipid droplet accumulation in NSCLC cells

**Figure 3:**
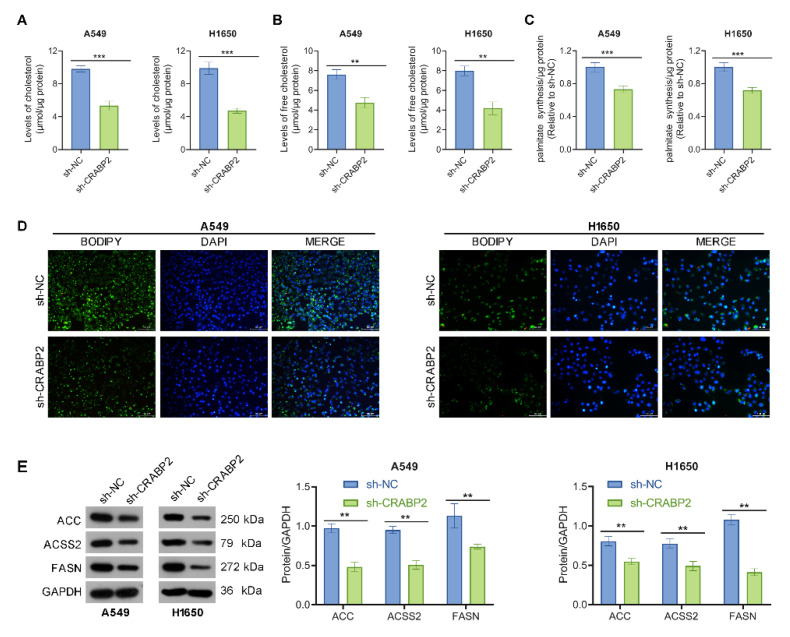
Downregulate of CRABP2 inhibits lipid content in NSCLC cells. (A) Intracellular levels of cholesterol were measured in A549 and H1650 cells with CRABP2 knocked down (n = 3). (B) Intracellular levels of free cholesterol were measured in A549 and H1650 cells with CRABP2 knocked down (n = 3). (C) Intracellular levels of palmitate were measured in A549 and H1650 cells with CRABP2 knocked down (n = 3). (D) Lipid droplets were visualized using Bodipy staining in A549 and H1650 cells with CRABP2 knocked down (n = 3). (E) Protein expression of ACC, ACSS2, and FASN in NSCLC cells was determined by Western blot analysis. **P < 0.01, ***P < 0.001.

Earlier research has highlighted the role of CRABP2 in the regulation of lipid metabolism 25, which is vital for cancer cell proliferation, invasion, and metastasis 26. Thus, we explored whether CRABP2 modulates lipid metabolism. As demonstrated in Figs.3A-B, the levels of total cholesterol and free cholesterol were decreased in shCRABP2 cells compared with those in control cells. In addition, the palmitate level was also decreased in shCRABP2 cells compared with those in control cells (Fig. 3C). BODIPY staining showed that CRABP2 knockdown inhibited the accumulation of lipid droplets in A549 and H1650 cells (Fig. 3D). In parallel, Western blot analysis revealed that the expression levels of ACC, ACSS2, and FASN were lower in shCRABP2 cells compared to control cells in both A549 and H1650 cells (Fig. 3E). In conclusion, our findings indicate that CRABP2 promoted lipid droplet accumulation.

### CRABP2 binds to PLAAT4 and decreases its protein stability

**Figure 4:**
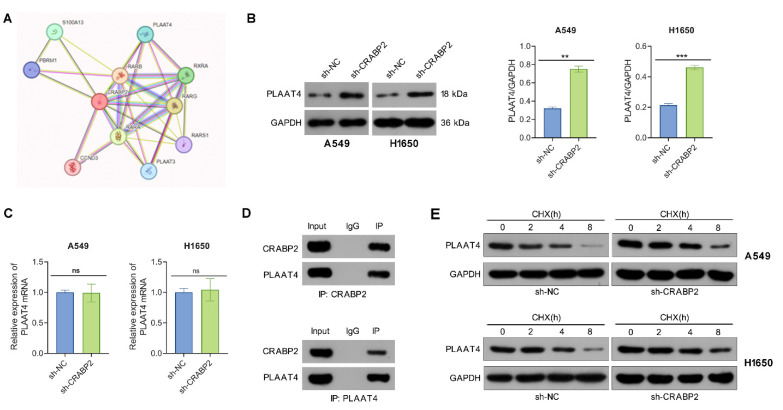
CRABP2 binds to PLAAT4 and promotes its degradation. (A) STRING predicts proteins that interact with CRABP2. (B) Western blot analysis was used to determine PLAAT4 protein expression following CRABP2 knockdown. (C) qRT-PCR were utilized to determine PLAAT4 mRNA expression following CRABP2 knockdown. (D) Co-immunoprecipitation (Co-IP) analysis demonstrating the interaction between CRABP2 and PLAAT4. The upper panel shows the results of the IP using CRABP2 antibody, while the lower panel presents the IP with PLAAT4 antibody. IgG serves as a negative control, and Input acts as a positive control. (E) Following cycloheximide (CHX) treatment, CRABP2-knockdown (shCRABP2) or control cells were collected at 0, 2, 4 and 8 h to assess the protein stability of CRABP2 and PLAAT4 via Western blotting. ns, no significance. *P < 0.05, ***P < 0.001.

The PPI network of CRABP2 was retrieved from the STRING database. Phospholipase A and acyltransferase 4 (PLAAT4), which is an enzyme involved in the synthesis of bioactive lipids, exhibited a high potential for interaction with CRBP2 and thus drew our attention (Fig. 4A). PLAAT4 belongs to the lecithin retinol acyltransferase (LRAT) protein family and has been proposed to suppress cancer cell invasion and metastasis. To determine if PLAAT4 is a downstream target of CRABP2, we first assessed whether CRABP2 influences the expression of PLAAT4.

The results from Western blot indicated that the protein levels of PLAAT4 in the sh-CRABP2 group were significantly elevated compared to those in the control group (Fig. 4B), although no differences were observed at the mRNA level as shown by qRT-PCR (Fig. 4C). Additionally, Co-IP experiments demonstrated that PLAAT4 co-immunoprecipitated with CRABP2 (Fig. 4D). To further explore how CRABP2 affects PLAAT4 protein levels, A549 and H1650 cells were treated with CHX. The findings revealed that the degradation rate of PLAAT4 protein was notably lower in the sh-CRABP2 group than in controls (Fig. 4E).

### Downregulation of PLAAT4 reverses reduced metastasis and lipid droplet induced by shCRABP2 in NSCLC cells

**Figure 5:**
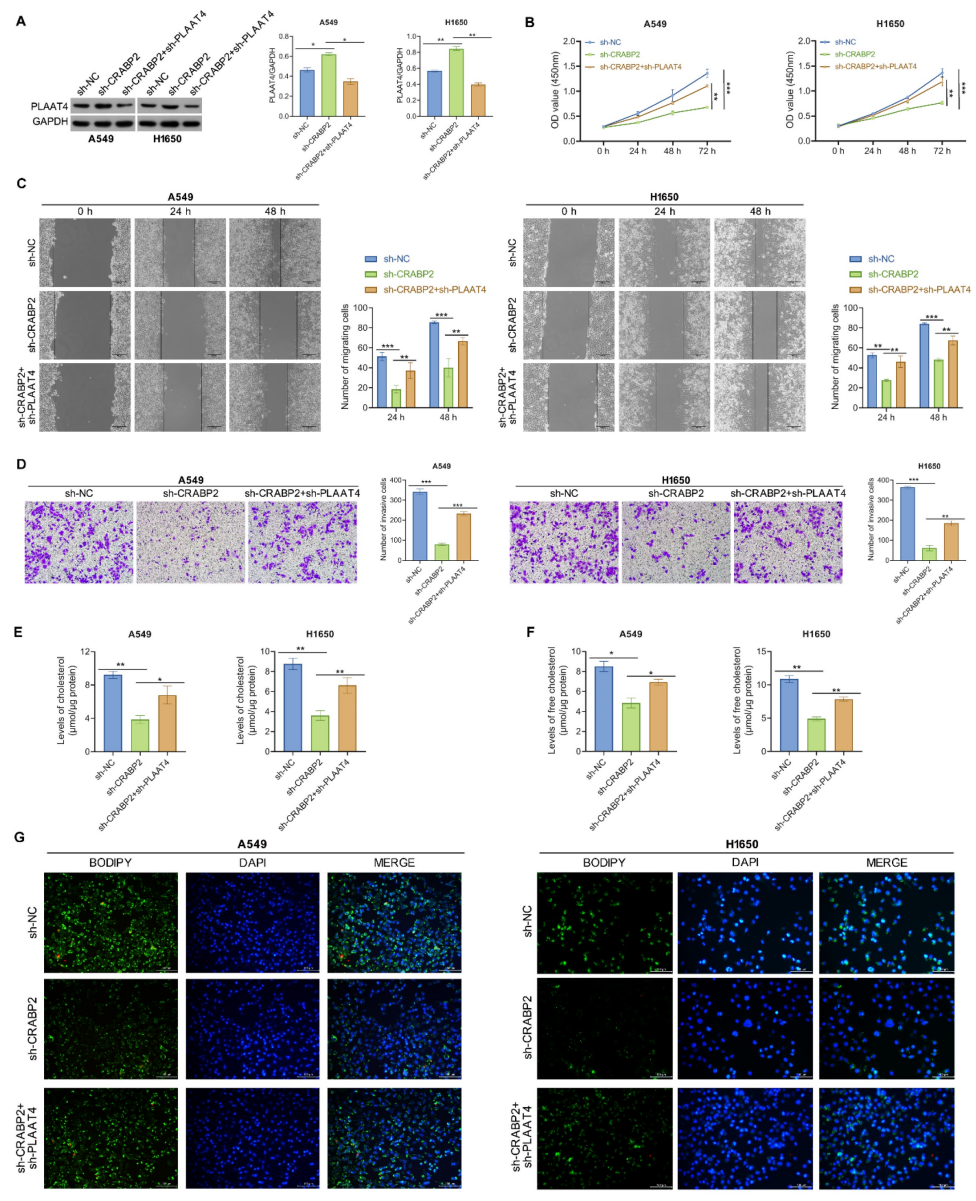
Downregulation of PLAAT4 reverses reduced metastasis and lipid droplet induced by shCRABP2 in NSCLC cells. (A) Protein expression of PLAAT4 was detected by Western blot analysis in NSCLC cells subjected to indicated transfection, including sh-NC, sh-CRABP2, and sh-CRABP2+sh-PLAAT4. (B) The proliferative ability was evaluated by CCK-8 in A549 and H1650 cells following the indicated treatments. (C) Wound-healing assays were employed to evaluate the migratory ability of A549 and H1650 cells subjected to the indicated treatment. (D) Transwell invasion assays were conducted to assess the invasive capabilities in A549 and H1650 cells subjected to the indicated treatment. (E) Levels of cholesterol were determined in A549 and H1650 cells with the indicated treatment. (F) Levels of free cholesterol were determined in A549 and H1650 cells with the indicated treatment. (G) Bodipy staining was performed to visualize lipid droplets in A549 and H1650 cells with the indicated treatment. *P < 0.05, **P < 0.01, ***P < 0.001; One-way or two-way ANOVA.

Then, functional rescue experiments were conducted to validate the impact of CRABP2 on lipid metabolism and metastasis of NSCLC cells through PLAAT4. Western blotting analysis demonstrated an increase in PLAAT4 expression in the shCRABP2 group, which was then suppressed upon PLAAT4 knockdown (Fig. 5A).

Celluar experiments indicated a significant reduction in cell proliferation, migration, and invasion following CRABP2 knockdown, while silencing of PLAAT4 effectively reversed these effects (Figs. 5B-D). Concurrently, it was observed that the lipid metabolic changes induced by shCRABP2 could be counteracted by silencing PLAAT4 (Figure 5E, F). Overall, these findings highlight the essential function of CRABP2 in enhancing lipid biosynthesis and facilitating metastatic potential of NSCLC cells through the downregulation of PLAAT4.

### CRABP2 accelerates tumor growth and metastasis by regulating the expression of PLAAT4 *in vivo*

**Figure 6:**
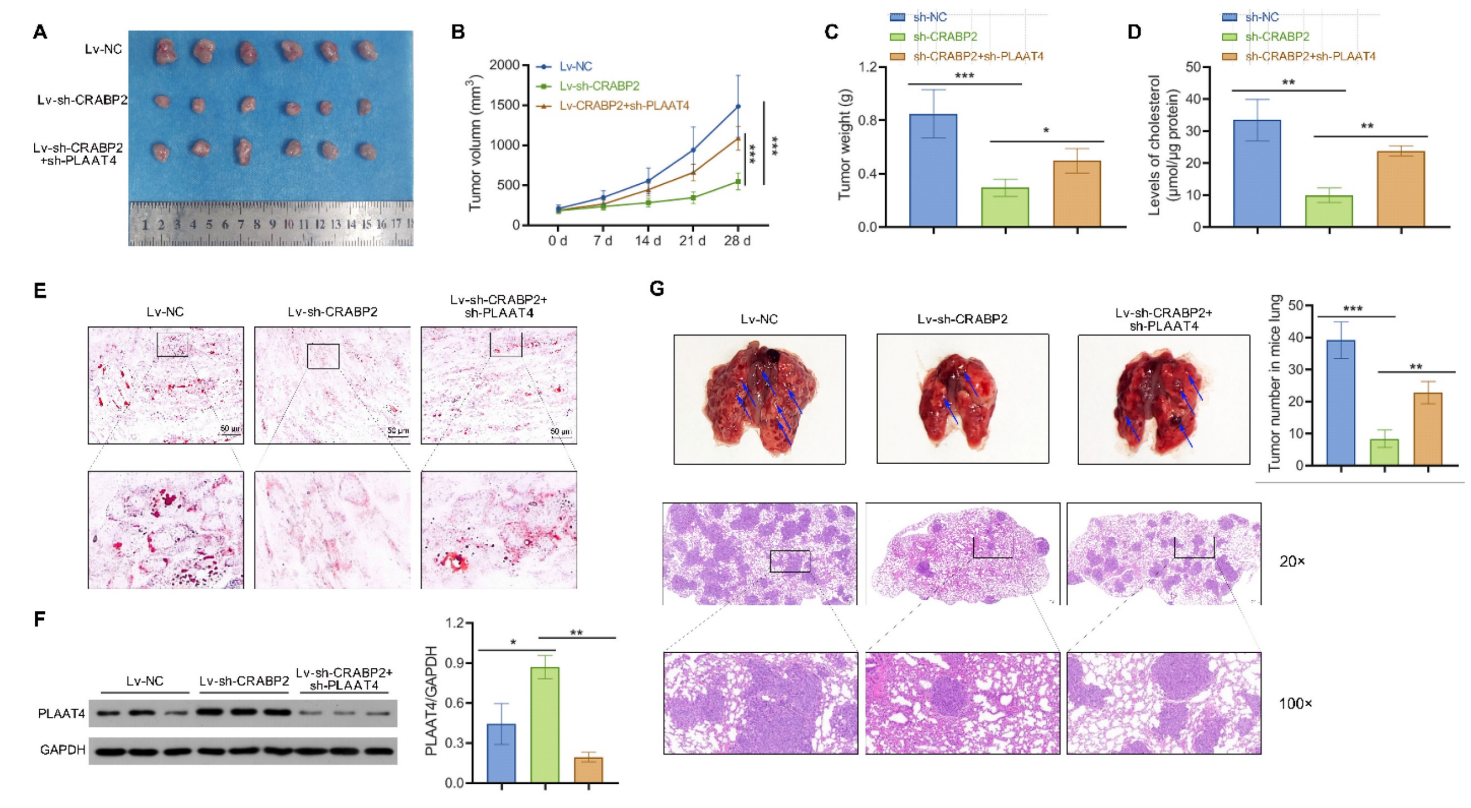
Targeting CRABP2 suppresses tumor growth and metastasis by enhancing PLAAT4 expression *in vivo*. (A) A549 cells were transfected with Lv-NC, Lv-sh-CRABP2, or Lv-sh-CRABP2+sh-PLAAT4, and subsequently injected subcutaneously into the flanks of 5-week-old male BALB/c nude mice (n = 6). Photographs of excised tumors were presented. (B) Growth curves of these tumors in tumor-bearing nude mice. (C) The average tumor weight of each group. (D) Cholesterol levels were detected in tumor tissues. (E) Lipid droplet content was assessed using Oil Red O staining in tumor samples. Scale bars = 50 μm. (F) Western blot analysis of PLAAT4 protein expression in tumor tissues. (G) A549 cells were stably transfected with Lv-NC, Lv-sh-CRABP2, or Lv-sh-CRABP2+sh-PLAAT4 and subsequently injected into nude mice via tail vein (n = 6). The formation of lung metastases was observed two months later and the blue arrows indicate representive metastases (upper). Paraffin embedding of lung tissue for HE staining analysis (lower). Scale bars = 500 μm. The number of lung metastases was counted. *P < 0.05, **P < 0.01; ***P < 0.001; One-way ANOVA.

To investigate the role of PLAAT4 in the phenotypes of NSCLC cells regulated by CRABP2, we generated CRABP2-KD alone or CRABP2/PLAAT4 double-KD A549 cells, and subcutaneously injected into nude mice (Fig. 6A). The reduction in cell growth caused by CRABP2 silencing was partially mitigated upon the deletion of PLAAT4 (Fig. 6B). In addition, the effect of reducing tumor weight by downregulating CRABP2 was significantly restored after silencing PLAAT4 (Fig. 6C). Moreover, CRABP2 depletion induced a decrease in levels of cholesterol that was restored to the normal control by further PLAAT4 KD (Fig. 6D). Nile Red staining also confirmed this finding (Fig. 6E). Western blotting analysis showed that the expression of PLAAT4 was increased in the shCRABP2 group, and inhibited by knockdown of PLAAT4 (Fig. 6F).

To assess the potential of CRABP2 to facilitate metastasis *in vivo*, A549-shCRABP2, and control cells were inoculated into nude mice via tail vein injection. After a period of 2 months, the mice were sacrificed for examination of metastatic nodules present in their lungs. H&E staining revealed a noteworthy decrease in the overall count of metastatic nodules in their lungs following CRABP2 deletion; however, this inhibitory effect could be partially restored by PLAAT4 knockdown (Fig. 6G). These findings suggested that CRABP2 facilitates tumor growth and metastasis of NSCLC by inhibiting PLAAT4 expression.

## Discussion

Primary lung cancer, with 80% being the NSCLC subtype, represents the leading cause of cancer-related fatalities globally. The metastasis process significantly contributes to the mortality in NSCLC patients 27. Therefore, gaining a comprehensive understanding of the metastatic process is essential for developing therapeutic approaches and enhancing patient outcomes. In this study, we analyzed RNA sequencing data of LUAC and normal tissues, identifying CRABP2 as a target gene that shows significant upregulation in LUAC. Importantly, our results show a strong correlation between CRABP2 levels and overall survival in LUAC patients, aligning with findings from a previous meta-analysis 23.

The role of CRABP2 has been explored in various cancers, emphasizing its involvement in tumor progression, metastasis, and drug resistance 22, 28, 29. For instance, in prostate cancer, CRABP2 enhances prostate cancer cell migration and invasion 17. In ovarian cancer, CRABP2 promotes tumorigenesis and olaparib resistance through downregulation of caspase-8 while decreasing reactive oxygen species production 18. Likewise, in gastric cancer, CRABP2 impedes mitochondrial apoptosis and diminishes sensitivity of oxaliplatin 19. Consistent with these findings, our results show that silencing CRABP2 inhibits NSCLC cell growth and metastasis in both *in vitro* and *in vivo* models. These findings reinforce the notion that CRABP2 is a critical factor in the progression of NSCLC.

Abnormal lipid metabolism is increasingly recognized as a significant metabolic alteration in various cancers, including NSCLC cancers 30. Studies have shown that lipids are involved in cell proliferation, apoptosis, drug resistance and other cellular behaviors in NSCLC. The research from Zhou Y et al. 31 showed that STRA6 enhances SREBP-1-mediated adipogenesis, providing energy for the growth of NSCLC cells.

Another study revealed that HIF1A activates PCDH7 to inhibit LUAD anoikis by facilitating fatty acid synthesis and metabolism 32. In addition, cholesterol has been shown to contribute to resistance against EGFR-TKIs in NSCLC 33. A hepatisis-related study has indicated that CRABP2 can promote lipid droplet accumulation 25, yet its effect on lipid metabolism in cancer cells have not been thoroughly characterized. In this study, we found that CRABP2 knockdown significantly reduced intracellular cholesterol, free cholesterol and palmitate levels, as well as lipid droplet accumulation. Notely, CRABP2 knockdown down-regulated lipid-related genes including ACSS2, ACC, and FASN. *In vivo* models, targeting CRABP2 also reduced lipid droplet as well. Overall, these findings revealed the promotive effect of CRABP2 on lipid droplet accumulation in NSCLC. Given the established role of lipid metabolism in cancer progression, we concluded that CRABP2 may regulate the lipid metabolic pathways of NSCLC cells to support growth and metastasis.

CRABP2 is a small and highly conserved protein known for its ability to bind to various proteins. Initial studies indicated that CRABP2 functions as a cytoplasmic shuttle protein, facilitating the transport of RA from the cytoplasm to the nucleus. In the nucleus, it delivers RA to RAR/RXR complexes, which subsequently activates gene expression 34. Subsequent research revealed that CRABP2 binds to and stabilizes the RNA-binding protein HuR, thereby enhancing the expression of transcripts dependent on HuR 35. Furthermore, Pastok et al. 36 identified CRABP2 as a specific binding partner for cyclin D3, which was later shown to promote RA-mediated transactivation of target genes through its interaction with both CRABP2 and RAR. In drug-resistant ovarian cancer cells, it was observed that CRABP2 increases HIF1α expression levels and enhances its nuclear localization 37. These findings reveal the ability of CRABP2 to interact with other proteins, shedding light on its diverse and important roles in cellular regulation and tumor biology. In this study, we confirmed that CRABP2 interacts with PLAAT4, influencing its protein stability and expression. The mechanism by which CRABP2 modulates PLAAT4 stability may involve the formation of a protein complex that induces PLAAT4 degradation through the proteasomal pathway or by inhibiting its translational efficiency. PLAAT4 was discovered to act as a tumor suppressor 38. For instance, Wei L. et al. 39 demonstrated the epigenetic silencing of PLAAT4 mediated by histone methyltransferase G9a promotes the development of liver cancer. In estrogen receptor-negative breast cancer, the absence of PLAAT4 is recognized as a significant factor contributing to lung metastasis by enhancing the adhesion of cancer cells to the lung stroma 40.

Additionally, PLAAT4 is an enzyme involved in lipid metabolism, particularly in phospholipid metabolism 41. It possesses phospholipase A1/A2 and acyltransferase activities, capable of catalyzing various reactions in phospholipid metabolism. For example, PLAAT4 has been shown to mediate the transfer of an acyl chain from glycerophospholipids, primarily phosphatidylcholine (PCs), to the amino group of the phosphatidylethanolamine (PEs), producing N-acylphosphatidylethanolamine (NAPE) that serves as the precursor for N-acylethanolamines (NAE) 38. Research from Liu SS et al reveals the significant connection of PLAAT4 in regulating the balance between lipid metabolism and antiviral immune responses. Given that the crucial roles of PLAAT4 in tumor progression and lipid metabolism, we hypothesized that it plays a vital role in the oncogenic activity of CRABP2. Subsequently, our study demonstrated that inhibiting PLAAT4 could reverse the inhibitory effects of sh-CRABP2 on migration, invasion, and lipid metabolism, indicating that CRABP2 promotes NSCLC progression via regulating PLAAT4.

## Conclusions

This study uncovers a novel CRABP2/PLAAT4-mediated lipid metabolic axis as the first reported mechanism driving NSCLC progression, where CRABP2 directly binds to PLAAT4 to decreases its protein stability, thereby enhancing lipid droplet accumulation and malignant phenotypes. These findings suggest that targeting CRABP2 may provide a promising approach to improving the clinical outcomes of this aggressive cancer type.

## Figures and Tables

**Table 1:** Correlation between CRABP2 expression and clinicopathological characteristics of NSCLC patients.

Characteristics	CRABP2		*P*-value
	High no. cases [%]	Low no. case [%]	
Age (years)			0.370
>60	7 (63.6%)	4 (36.4%)	
≤60	3 (33.3%)	6 (66.7%)	
SEX			1.000
Male	6 (50.0%)	6 (50.0%)	
Female	4 (50.0%)	4 (50.0%)	
Smoke			0.582
No	7 (43.8%)	9 (56.3%)	
Yes	3 (75.0%)	1 (25.0%)	
Clinical stage			0.170
I/II	4 (33.3%)	8 (66.7%)	
III/IV	6 (75.0%)	2 (25.0%)	
Metastasis			0.074
Negative	3 (30.0%)	7 (70.0%)	
Positive	7 (70.0%)	3 (30.0%)	
